# Psychometric Properties of Instruments Measuring Health Sciences Students' Perceptions of Objective Structured Clinical Examinations: A Systematic Review

**DOI:** 10.1002/nop2.70341

**Published:** 2025-11-19

**Authors:** Seda Güney, Seda Karakaya Çataldaş, Tuba Sengul

**Affiliations:** ^1^ Faculty of Nursing Koç University Istanbul Türkiye

**Keywords:** educational measurement, objective structured clinical examination (OSCE), perception, psychometrics, students, systematic review

## Abstract

**Aim:**

To investigate the instruments used to measure students’ perceptions of the Objective Structured Clinical Examination (OSCE) in health sciences and to evaluate their methodological quality.

**Design:**

A systematic review of the peer‐reviewed published literature was conducted to identify, appraise, and summarize the characteristics and methodological quality of instruments developed to assess students’ perceptions of the OSCE in health sciences education. The review followed the PRISMA guidelines to ensure methodological transparency and reproducibility. The MERSQI checklist was applied to check the quality of the articles.

**Methods:**

Seven electronic databases were systematically searched for studies published between January 2014 and May 2025. Manual reference searches were also performed to capture additional relevant studies. Studies were included if they reported primary research describing the development, adaptation, or psychometric evaluation of instruments measuring students’ perceptions of OSCE experiences in health sciences programs. Methodological quality was assessed using the MERSQI checklist, and studies scoring below 60 out of 100 were excluded evaluate the quality of the included studies, focusing on study design, sampling, data analysis, validity, and outcomes.

**Results:**

A total of 1,578 records were screened, with only two studies meeting the inclusion and quality criteria. The majority of identified instruments lacked psychometric rigor, as they were author‐developed questionnaires without formal reliability or validity testing. Two validated instruments were identified: the SINE‐OSCA Scale and the OSCEPS. The SINE‐OSCA demonstrated acceptable psychometric properties, while the OSCEPS exhibited excellent internal consistency and acceptable structural validity, supporting its use for evaluating OSCE experiences.

**Conclusion:**

These findings underscore the scarcity of validated instruments and emphasize the need to test and develop psychometrically robust tools to support assessment practices in OSCE‐based education.

**Patient or Public Contribution:**

No Patient or Public Contribution.

## 1 Introduction

Dr. Ronald Harden developed the Objective Structured Clinical Examination (OSCE) in the 1970s to assess clinical competence through a structured and objective approach (Bartfay et al. 2004). It has become widely used in health education, particularly in medical and nursing programs, for evaluating essential professional skills in realistic clinical environments (Taghva, et al. 2010; Ahmed et al. 2015). OSCE's structured tasks simulate clinical scenarios, helping students develop and refine their clinical skills (Ahmed et al. 2015). Studies have confirmed its reliability and validity in assessing diverse clinical skills (Nickbakht et al. 2013; Smith et al. 2012; Taghva, et al. 2010), and it is an integral part of curriculum planning (Duerson et al. 2000; Vincent et al. 2022). Although the effectiveness of this method has been proven, research on the validity and reliability of the instruments used to measure students' perceptions of OSCE needs to be more comprehensive (Majumder et al. 2019). A key concern is that instruments without rigorous psychometric evaluation may distort students' experiences and competencies, affecting their readiness for clinical practice (Okugbo et al. 2020; Jawaid et al. 2014; Montgomery et al. 2021).

Previous studies have examined students' attitudes, satisfaction and feedback regarding OSCEs (Arekat et al. 2022; Chabrera et al. 2023; Goh et al. 2016), yet the psychometric properties of these instruments still need to be fully explored. Evaluating these assessment tools' psychometric properties, such as reliability, validity and internal consistency, is critical to accurately interpreting student outcomes. Good psychometric properties typically include internal consistency (e.g., Cronbach's alpha ≥ 0.70), construct validity demonstrated through exploratory or confirmatory factor analysis, and test–retest reliability (Elsman et al. 2024). The Consensus‐based Standards for the selection of health Measurement Instruments (COSMIN) framework provides a robust methodology for this evaluation (Mokkink et al. 2010; Elsman et al. 2024). Despite its importance, existing reviews reveal significant gaps in the comprehensive validation of instruments assessing OSCE perceptions, with many tools lacking thorough psychometric evaluation (Torabizadeh et al. 2018; Sloan et al. 1995). Despite the growing literature, few instruments have undergone rigorous psychometric testing. In this review, 1578 records were screened, but only two validated instruments met the inclusion and methodological quality criteria (MERSQI ≥ 60). This substantial gap highlights the limited availability of methodologically sound tools for evaluating student perceptions of OSCE. Recent studies have begun to address this need by developing instruments grounded in strong psychometric principles to enhance the reliability and validity of student feedback in OSCE contexts.

## 2 Methods

### 2.1 Research Questions

Is there a well‐developed tool with psychometric properties to evaluate OSCE through student perception, satisfaction, experiences and opinions in health sciences?

### 2.2 Study Design

Following the Preferred Reporting Items for Systematic Reviews and Meta‐Analyses (PRISMA) guidelines (Page, McKenzie, et al. 2021), a systematic review was conducted to evaluate and synthesise research articles that examine the development of scales assessing the perceptions of health sciences students. The PRISMA framework ensures a transparent and comprehensive approach to reviewing the literature, enhancing the reliability and replicability of findings. This review aimed to identify studies that developed, validated, or evaluated the psychometric properties of instruments used to measure students' perceptions of the Objective Structured Clinical Examination (OSCE) in health sciences education. By adhering to PRISMA guidelines, the review included a structured process of literature search, study selection, data extraction and synthesis, providing an evidence‐based overview of the quality and scope of the existing tools used in OSCE perception assessments.

### 2.3 Eligibility Criteria

The study keywords were selected according to the Medical Subject Headings and PICOS. The inclusion criteria for the studies according to the Patients Interventions Comparison Outcome Time‐Study Design (PICOT‐SD) are as follows.

1. Participants (P): Medicine, nursing and health sciences students.
2. Intervention (I): Instruments (scales, tools) used to evaluate students' perceptions of OSCE, with psychometric properties such as test–retest reliability, Kappa, Exploratory Factor Analysis (EFA), Confirmatory Factor Analysis (CFA), validity and other related measures.
3. Comparisons (C): Comparisons of instruments with different levels of psychometric validation (e.g., fully validated vs. partially validated or non‐validated tools).
4. Outcomes (O): Reported psychometric outcomes, including validity, reliability, test–retest reliability, Kappa, EFA, CFA and other relevant statistics.
5. Time (T): During students' education in health sciences programs.
6. Setting (S): Educational institutions offering health sciences programs.
7. Study Design (SD): Randomised controlled trials, descriptive, experimental, quasi‐experimental and qualitative studies.

### 2.4 Search Strategy

Articles were retrieved from seven databases: PubMed, Cochrane Library, Medline (OVID), Scopus, Web of Science, CINAHL and Science Direct.

The search strategy included the following Boolean terms and keywords: (“Objective Structured Clinical Examination” OR “OSCE”) AND (“psychometric testing” OR “validity” OR “reliability” OR “development testing”) AND (“assessment” OR “feedback” OR “student satisfaction” OR “perception” OR “attitude” OR “experience”) AND (“nursing” OR “medicine” OR “health sciences”). Search strings were adapted per database with both controlled vocabulary (e.g., MeSH) and free‐text terms to maximise comprehensiveness.

A health sciences librarian was consulted to ensure search precision and completeness. All records were imported into COVIDENCE for screening and data extraction. Covidence is a web‐based collaboration software platform that streamlines the production of systematic and other literature reviews (Covidence 2025). Duplicate records were automatically removed and the remaining articles were screened independently by two reviewers based on the inclusion and exclusion criteria.

### 2.5 Inclusion and Exclusion Criteria

Studies were included based on the following criteria:

1. Evaluation tools/instruments of OSCE by medical and nursing students.
2. Participants were medical and nursing faculty students.
3. Full‐text and peer‐reviewed articles in English.
4. Articles published between January 1, 2014 and May 26, 2025.
5. Instruments that were developed or used in the study with proper psychometric properties (e.g., internal consistency, construct validity—exploratory and confirmatory factor analysis, and reliability).
6. Additionally, articles that addressed general aspects of OSCE and those that evaluated online OSCEs were excluded.
7. Furthermore, the systematic review did not include studies published in predatory journals, those without study protocols, those with insufficient results, non‐English studies, unpublished studies and pilot studies.

### 2.6 Study Selection and Data Extraction

All three investigators reviewed this table. In the first step, one researcher (S.G.) imported the references into Covidence and removed duplicates. The initial screening round, focusing on titles and abstracts, was conducted by two investigators (S.K.C. and S.G.). Subsequently, three investigators (S.G., T.S. and S.K.C.) independently reviewed the selections to identify full‐text articles that met the inclusion and exclusion criteria. Any disagreements were resolved through consensus among all authors. In the next phase, all researchers (T.S., S.K.C., and S.G.) independently reviewed the full texts of the selected articles and made the final decisions on inclusion. In cases of disagreement during title/abstract or full‐text screening, consensus was reached through discussion. When necessary, a third reviewer (S.K.C.) was consulted to make the final decision. Predatory journals were excluded if they met two or more indicators, such as the absence of a transparent editorial board, unverifiable or misleading impact metrics, and excessive publication fees without a clearly documented peer review process. The screening of predatory publications was further supported using Cabell's Blacklist (http://cabells.com). Data extraction was guided by a pre‐defined template developed in Covidence to ensure consistency across reviewers. Key variables included study author(s), year, country, sample size, study design, type of tool, psychometric properties and main outcomes (as detailed in Table 1). All three investigators reviewed and cross‐checked the extracted data to ensure accuracy and completeness.

**TABLE 1:** Data extraction.

Author, year, country	Study design	Sample	Scale/Questionnaire	Psychometric properties of the instrument	Quality appraisal‐MERSQI score	Outcomes
Hunt et al. 2020, Australia	Methodological study	Final year students (*n* = 727) enrolled in an undergraduate nursing program	Scale items were related to assessment fairness, a reflection of students' ability, the usefulness of OSCA guidelines, students' confidence and time for assessment preparation was included. Each item has a 7‐point Likert scale response format that ranged from strongly disagree (1) to strongly agree (7). The possible aggregate scores of the SINE‐OSCA ranged from 10 to 70	Exploratory factor analysis was conducted: Principal Axis Factoring, which accounted for 51% of the variance. Factor loadings of all 10 items ranged from 0.45 to 0.86. Cronbach's alpha coefficient of the one‐factor SINE‐OSCA was 0.91	69.5	The SINE‐OSCA scale demonstrates validity and reliability in identifying students who may have difficulty with this mode of clinical skill assessment
Sengul et al. 2024, Türkiye	Methodological study	Medical and nursing students (*n* = 220) from two faculties	OSCEPS; a 19‐item, one‐dimensional 5‐point Likert‐type scale developed to evaluate health sciences students' perceptions of the OSCE. Items address organisation, realism, clarity of instructions, assessment fairness and alignment with learning objectives. Scores range from 19 to 95; higher scores indicate more positive perceptions	Content Validity Index (S‐CVI = 0.97, I‐CVI = 0.91); Exploratory Factor Analysis explained 56.17% of total variance; KMO = 0.944, Bartlett's test *p* < 0.001; Confirmatory Factor Analysis fit indices: RMSEA = 0.084, CFI = 0.902, TLI = 0.889; Cronbach's alpha = 0.943; Test–retest reliability: *r* = 0.799, *p* < 0.001; item‐total correlations ranged from 0.593 to 0.804	60	The OSCEPS demonstrated strong validity and reliability in evaluating health sciences students' perceptions of OSCE

### 2.7 Appraisal of Methodological Quality

Three reviewers independently assessed the quality of the studies included in this review using a modified version of the Medical Education Research Study Quality Instrument (MERSQI), a validated and widely used tool in medical education research (Smith and Learman 2017). MERSQI evaluates seven domains: study design (23 points), sampling (10 points), setting (8 points), type of data (11 points), validity of the evaluation instrument (15 points), data analysis (17 points) and outcomes (16 points). The total possible score is 100 in the quality checklist. To ensure consistent interpretation and facilitate comparison across studies, the authors categorised study quality as follows: low (< 45), moderate (45–60) and high (> 60). Studies scoring below 60 points were excluded from the review. MERSQI scoring was independently performed by two trained reviewers and inter‐rater reliability was calculated using the intraclass correlation coefficient (ICC = 0.82), indicating excellent agreement.

## 3 Results

### 3.1 Is There a Tool That Evaluates OSCE Through Student Perception, Satisfaction, Experiences and Opinions in Health Sciences?

**FIGURE 1:**
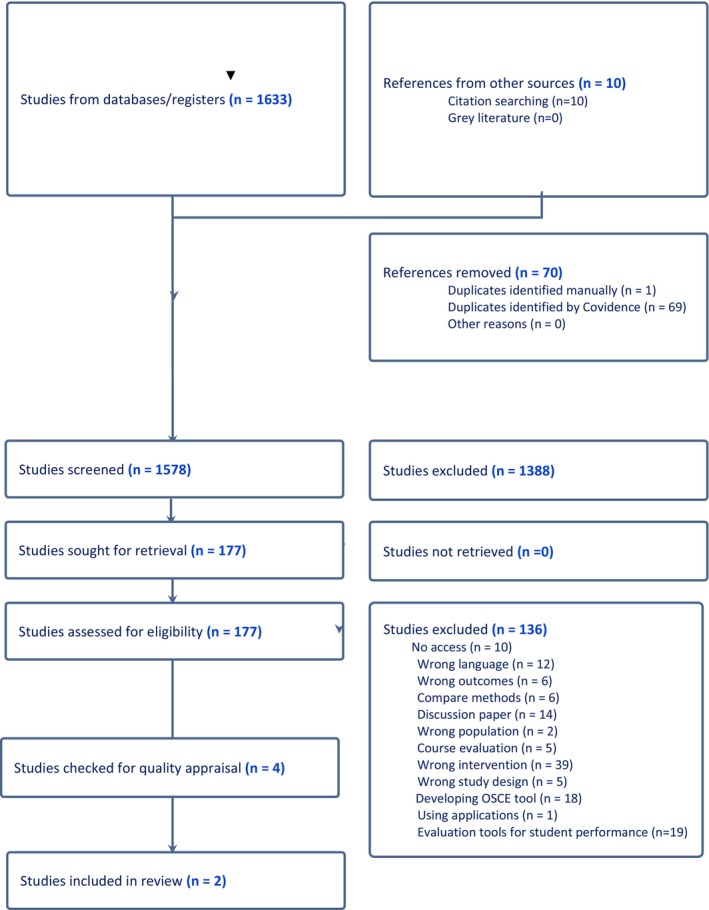
PRISMA. *Source:* Page, Moher, et al. (2021).

Initially, 1633 articles were identified through database searches, and 10 additional records were obtained via manual reference screening. After removing duplicates, 177 articles remained for title and abstract screening. Of these, 41 articles met the inclusion criteria for full‐text review. Many of the excluded studies either lacked psychometric evaluation, used non‐validated tools, or failed to provide sufficient methodological detail. Figure 1 presents the PRISMA diagram outlining the selection process and reasons for exclusion. All 41 studies were appraised for methodological quality using the MERSQI checklist by three independent reviewers. Only two studies scored above the threshold of 60 out of 100 and were included in the final synthesis (Table 1). This finding emphasises the limited availability of rigorously validated instruments to assess student perceptions of OSCE in health sciences education. Most of the reviewed instruments were author‐developed questionnaires without standardised psychometric validation. A substantial portion of these studies relied on the original questionnaire developed by Pierre et al. (2004), which lacks comprehensive validity and reliability testing. This tool was adapted in various studies, typically ranging between 16 and 26 items, and was often supported only by descriptive statistics or internal consistency coefficients such as Cronbach's alpha (El‐Sheikh and Abd El Aziz 2015; Nafee et al. 2019; Ahmed et al. 2015). Similarly, other tools used across studies also lacked robust psychometric analysis, including those by Muldoon et al. (2014), Badur et al. (2017), Rao et al. (2014), Labaf et al. (2014) and Soni et al. (2017).

### 3.2 Do These Measurement Tools Demonstrate Methodological Soundness?

The included studies were independently assessed for methodological quality by the authors, focusing on criteria such as scale development, content validity, structural validity, internal consistency and reliability testing. Among the 41 studies reviewed, only two instruments met the minimum MERSQI threshold of 60 points.

The first tool, the Satisfaction with Nursing Skill Examination—Objective Structured Clinical Assessment (SINE‐OSCA), underwent comprehensive psychometric testing. It consists of 10 items on a 7‐point Likert scale (1 = strongly disagree to 7 = strongly agree) and was designed to assess students' satisfaction and perceptions of OSCE. Exploratory factor analysis revealed a one‐factor solution accounting for 51% of the variance, with factor loadings ranging from 0.45 to 0.86, well above the commonly accepted threshold of 0.40. The internal consistency of the scale was excellent, with a Cronbach's alpha of 0.91. Corrected item‐total correlations exceeded 0.30 for all items, indicating strong item discrimination. The scale covers key areas such as perceived fairness, reflection of student ability, clarity of guidelines, confidence levels and time sufficiency for preparation (Hunt et al. 2020). The second validated tool, the Objective Structured Clinical Examination Perception Scale (OSCEPS), was also supported by robust psychometric evaluation, including confirmatory and exploratory factor analysis, and demonstrated excellent internal consistency (Cronbach's alpha = 0.943). This scale evaluated perceptions such as fairness, realism, feedback and alignment with learning outcomes (Sengul et al. 2024).

In contrast, the remaining 38 instruments provided only partial or preliminary psychometric testing. Some studies reported only content validity (Ahmed et al. 2015; Nafee et al. 2019), internal consistency via Cronbach's alpha (Muldoon et al. 2014), item‐total correlations (Labaf et al. 2014), or limited exploratory factor analysis without further validation (Müller et al. 2018). These findings highlight a lack of comprehensive methodological rigour in most of the available OSCE perception instruments.

## 4 Discussion

The assessment of OSCE through student perception, satisfaction, experiences and opinions in health sciences is crucial for evaluating the overall effectiveness of this assessment method. A validated OSCE perception tool is essential for obtaining standardised and comparable outcomes in healthcare education. It provides a structured way to gather student feedback, which helps evaluate how effective and well‐received the OSCE is as an assessment method. The OSCE is known for its ability to assess clinical skills consistently, and understanding students' views can guide educators in improving the exam process to support learning better (Ahmed et al. 2015; Alamri et al. 2022). The validity and reliability of a tool should be established across different populations and cultural contexts to ensure its robustness and adaptability. By validating the tool in diverse settings, outcomes can be synthesised and interpreted more effectively, providing a comprehensive understanding of OSCE's impact on student learning and clinical competence.

Despite the importance of the perception of students for the OSCE, no systematic reviews have addressed this issue. Thus, we aimed to fill this gap by synthesising existing literature on the psychometric properties of instruments that measure health sciences students' perceptions of OSCE. We identified 1633 articles through database searches and an additional nine articles found through manual reference searching. After screening and removing duplicates, 41 articles were selected for full‐text review, explicitly focusing on students' perceptions of health sciences.

Three independent reviewers assessed the methodological quality of the studies, focusing on the measurement properties of the tools used to evaluate student perceptions of OSCE, utilising the MERSQI checklist (Smith and Learman 2017). Notably, only two studies scored above 60 points, indicating high methodological quality, and were therefore included in the review. This result highlights the importance of maintaining rigorous methodological standards when evaluating student perceptions of OSCE in health sciences. The decision to include studies scoring 60 points or higher on the MERSQI checklist was based on the authors' decision to facilitate interpretation and comparison of methodological rigour across studies.

Most instruments used to evaluate student perceptions of OSCE are questionnaires developed by the authors, which have yet to undergo comprehensive psychometric validation processes. Many studies (Ahmed et al. 2015; Jawaid et al. 2014; Nafee et al. 2019) used the original questionnaire developed by Pierre et al. (2004) without conducting sufficiently thorough validity and reliability testing. The questionnaire developed by Pierre et al. consists of 32 items categorised into four sections, aiming to gather student feedback regarding various aspects of the OSCE experience, including its organisation, clarity and perceived effectiveness (Alghamdi et al. 2016). This questionnaire was used as a measurement to evaluate students' perceptions, 3, 23–24 (Ahmed et al. 2015; El‐Sheikh and Abd El Aziz 2015; Nafee et al. 2019), and only descriptive analysis, Cronbach's alpha, or content validity was reported. Similarly, other studies (Badur et al. 2017; Jawaid et al. 2014) used questionnaires developed by different researchers, which needed more adequate validation processes.

A measurement must be able to determine when any change is occurring. Not testing sensitivity to change raises doubts about a measurement's ability to assess the impact of interventions accurately. Most studies investigating the perceptions of health science students have reported only Cronbach's alpha (Llaguno et al. 2024; Yuan 2023), which indicates the reliability of the research but lacks factor analysis. Although one study (Labaf et al. 2014) reported an inter‐class correlation coefficient, it could not be included in our systematic review due to a score below 60 on the quality assessment. Conversely, the remaining instruments in the reviewed studies underwent limited validation processes, such as content validity assessment, Cronbach's alpha interpretation, item‐total correlation analysis, or exploratory factor analysis. These results indicate varying levels of methodological rigour in developing and validating tools for assessing student perceptions of OSCE in health sciences. This highlights the incomplete psychometric evaluation of tools used to assess student perceptions of OSCE and the need for scales with specific psychometric properties to evaluate OSCE, revealing a prevalent issue in the literature.

Among the identified instruments, only two tools underwent comprehensive psychometric evaluation. The first is the Satisfaction with Nursing Skill Examination: Objective Structured Clinical Assessment Scale (SINE‐OSCA), which demonstrated solid methodological rigour with strong internal consistency (Cronbach's *α* = 0.91) and satisfactory exploratory factor analysis for assessing students' satisfaction with OSCE implementation (Hunt et al. 2020). SINE‐OSCA consists of 10 items on a 7‐point Likert scale and measures components such as fairness, self‐confidence, clarity of guidelines and preparation time. These findings indicate that while some tools exhibit methodological soundness, a clear gap remains in the availability of validated and comprehensive instruments assessing student perceptions of OSCE.

The second tool, the Objective Structured Clinical Examination Perception Scale (OSCEPS), was recently published and validated through a study involving 220 nursing and medical students. Comprising 19 items, OSCEPS demonstrated excellent internal consistency (Cronbach's *α* = 0.943) and acceptable structural validity (RMSEA = 0.084; CFI = 0.902; TLI = 0.889). It evaluates key aspects of the OSCE experience, including fairness, clarity, realism, feedback and alignment with learning outcomes (Sengul et al. 2024). Rather than viewing this as a limitation, these findings serve as a benchmark for the field, illustrating what a well‐constructed and psychometrically sound OSCE perception tool can achieve. Their inclusion highlights not only the scarcity of high‐quality instruments but also the potential for future development based on established methodological standards. This review focused specifically on validated instruments due to the significant limitations observed in existing tools. Although numerous instruments exist to measure students' perceptions of OSCE, most lack robust validation, making it difficult to synthesise findings or recommend standardised use. The inclusion of only two studies in this systematic review highlights the urgent need for psychometrically sound tools to assess OSCE perceptions, which are essential for improving assessment practices, ensuring educational quality and fostering student‐centered clinical learning environments. Despite the rigorous selection process, our review was subject to certain limitations, as outlined below.

### 4.1 Limitations

This review has several limitations. Many studies identified during the screening process utilised instruments that lacked adequate psychometric validation, which restricted their inclusion. Additionally, the exclusion of non‐English publications may have limited the scope of the review by omitting potentially relevant tools developed in other languages or regions. Although the quality appraisal was conducted independently by trained reviewers, a degree of subjectivity remains inherent in such evaluations. Furthermore, while applying stringent inclusion criteria ensured methodological rigour, it may have reduced the breadth of included studies by excluding instruments that were in early stages of development or partial validation. These factors may affect the generalisability of the findings and highlight the need for further research to develop and validate tools in this field.

## 5 Conclusion

This systematic review comprehensively evaluated the reliability and validity of instruments used to assess health sciences students' perceptions of the OSCE. The findings indicate that while various questionnaires have been employed to measure student perceptions of the OSCE, their psychometric properties still need to be developed. Current tools require further refinement and ongoing development to ensure accurate and meaningful assessments. Future research should prioritise the creation of scales specifically designed to evaluate OSCE perceptions, focusing on rigorous psychometric testing. Furthermore, broader studies are essential to improve the generalisability of these instruments across diverse educational settings and student populations.

## Author Contributions

Substantial contributions to the conception or design of the work (S.G., T.S., S.K.C.); or the acquisition, analysis, or interpretation of data for the work (S.G., T.S., S.K.C.); drafting the work or revising it critically for important intellectual content (S.G., T.S., S.K.C.); final approval of the version to be published (S.G., T.S., S.K.C.); agreement to be accountable for all aspects of the work in ensuring that questions related to the accuracy or integrity of any part of the work are appropriately investigated and resolved (S.G., T.S., S.K.C.).

## Ethics Statement

This systematic review involves the collection and analysis of data from previously published studies. As the review did not involve direct contact with human participants or collecting new data, it was exempt from requiring formal ethics approval. All data used in this review were sourced from publicly accessible, peer‐reviewed literature, in accordance with the guidelines for ethical research practice. No identifying information or sensitive data about individuals was utilised in this study. Furthermore, the review adhered to ethical principles of research integrity, including transparency, accuracy and proper citation of all sources.

## Conflicts of Interest

The authors declare no conflicts of interest.

## Data Availability

The data that support the findings of this study are available on request from the corresponding author. The data are not publicly available due to privacy or ethical restrictions.
